# The role of neutrophil percentage to albumin ratio in predicting 1-year mortality in elderly patients with hip fracture and external validation

**DOI:** 10.3389/fimmu.2023.1223464

**Published:** 2023-08-09

**Authors:** Songsong Jiao, Jiangfei Zhou, Zhencheng Feng, Jian Huang, Lihong Chen, Zhiwu Li, Qingqi Meng

**Affiliations:** ^1^Department of Orthopedics, Guangzhou Red Cross Hospital, Jinan University, Guangzhou, China; ^2^Department of Traumatic Orthopaedics, The Central Hospital of Xiaogan, Xiaogan, Hubei, China; ^3^Department of Orthopedics, Bijie Second People’s Hospital, Guizhou, China

**Keywords:** neutrophil percentage to albumin ratio, hip fractures, neutrophil, albumin, prognosis

## Abstract

**Objectives:**

This study aimed to investigate the association between the neutrophil percentage to albumin ratio (NPAR) on the day of admission and mortality 1 year after surgery in elderly patients with hip fractures.

**Methods:**

Clinical characteristics and blood markers of inflammation were retrospectively collected from October 2016 to January 2022 in elderly patients with hip fractures at two different regional tertiary medical centers. It is divided into a training set and an external validation set. Multivariate Nomogram models such as NPAR were constructed using the least absolute shrinkage and selection operator (LASSO) regression results and multi-factor logistic regression analysis. In addition, multivariate Cox regression analysis and Kaplan-Meier survival curves were used to explore the relationship between NPAR values and mortality within 1 year in elderly patients with hip fractures. The predictive performance of the Nomogram was evaluated using the concordance index (C Index) and receiver operating characteristic curve (ROC) and validated by Bootstrap, Hosmer-Lemesow goodness of fit test, calibration curve, decision curve, and clinical impact curve analysis.

**Results:**

The study included data from 1179 (mean age, 80.34 ± 8.06 years; 61.4[52.1%] male) patients from the Guangzhou Red Cross Hospital affiliated with Jinan University and 476 (mean age, 81.18 ± 8.33 years; 233 [48.9%] male) patients from the Xiaogan Central Hospital affiliated with Wuhan University of Science and Technology. The results showed that NPAR has good sensitivity and specificity in assessing patients’ prognosis 1 year after surgery. Multivariate logistic regression models based on influencing factors such as NPAR have good discrimination and calibration ability (AUC=0.942, 95% CI:0.927-0.955; Hosmer-Lemeshow test: P >0.05). Kaplan-Meier survival curves for the training and validation sets showed that patients in the high NPAR group had a higher mortality rate at 1 year compared to the low NPAR group (P< 0.001). Multivariate Cox regression showed that high NPAR values were an independent risk factor for death within 1 year in elderly hip fracture patients (P< 0.001, HR =2.38,95%CI:1.84-3.08).

**Conclusion:**

Our study showed that NPAR levels were significantly higher in patients who died within 1 year after surgery in both the training and validation sets. NPAR has good clinical value in assessing 1-year postoperative prognosis in elderly patients with hip fractures.

## Introduction

Hip fractures are a common type of fracture in older adults. As the population ages, it is reported that there were 1.6 million hip fractures worldwide in 2000 and that this number will reach 3 million in 2050 (1). Patients with hip fractures are at high risk of death, with mortality rates as high as 15-36% 1 year after surgery (2–4). Hip fractures have become a worldwide public health problem of significant proportions due to their high morbidity and mortality. Therefore, predicting the risk of death in hip fracture patients in advance can help improve the prognosis by detecting and giving treatment in time (5). However, effective biological indicators that can adequately assess the postoperative prognosis of elderly hip fracture patients are lacking (6).

The relationship between inflammation and prognosis has received increasing attention in recent years. Studies have shown that (7–9) mortality 30 days after hip fracture is significantly associated with elevated levels of inflammatory markers, such as C-reactive protein (CRP), neutrophil-to-lymphocyte ratio (NLR), and ferritin. Neutrophils are key in protecting the body from various pathogenic microbial infections. At the same time, the neutrophil, an essential component of the innate immune system, is the first subset of immunoreactive cells to be attacked by antigens. Neutrophils can also activate other immune cells and induce cellular and humoral immunity (10, 11). Lymphocytes are essential for acquired immunity, providing broader and more precise recognition of antigens (12, 13). The percentage of neutrophils and lymphocytes often fluctuates from one to the other. Therefore, the neutrophil percentage not only directly reflects the ratio of neutrophils but also indicates the ratio of lymphocytes to a certain extent. Serum albumin is an important indicator of the patient’s nutritional status, and its level value is closely related to postoperative complications in patients (14). Neutrophil percentage to albumin ratio (NPAR) is a new marker of systemic inflammation that has been reported to be closely associated with the prognosis of many diseases, such as infectious shock (15), anti-NMDAR encephalitis (16) and bladder cancer (17). However, in different diseases, the NPAR value is different, and the proportion of deaths is also different. There are no clear reports on whether NPAR levels can predict the risk of postoperative mortality in elderly hip fractures.

Given that NPAR is an economical and easily accessible parameter of blood inflammation, understanding and confirming its predictive value would be very helpful in clinical decision-making. This study aimed to analyze and summarize NPAR levels in elderly patients with hip fractures and to investigate its role in assessing poor postoperative prognosis.

## Methods

### Participants and study design

**Figure 1:**
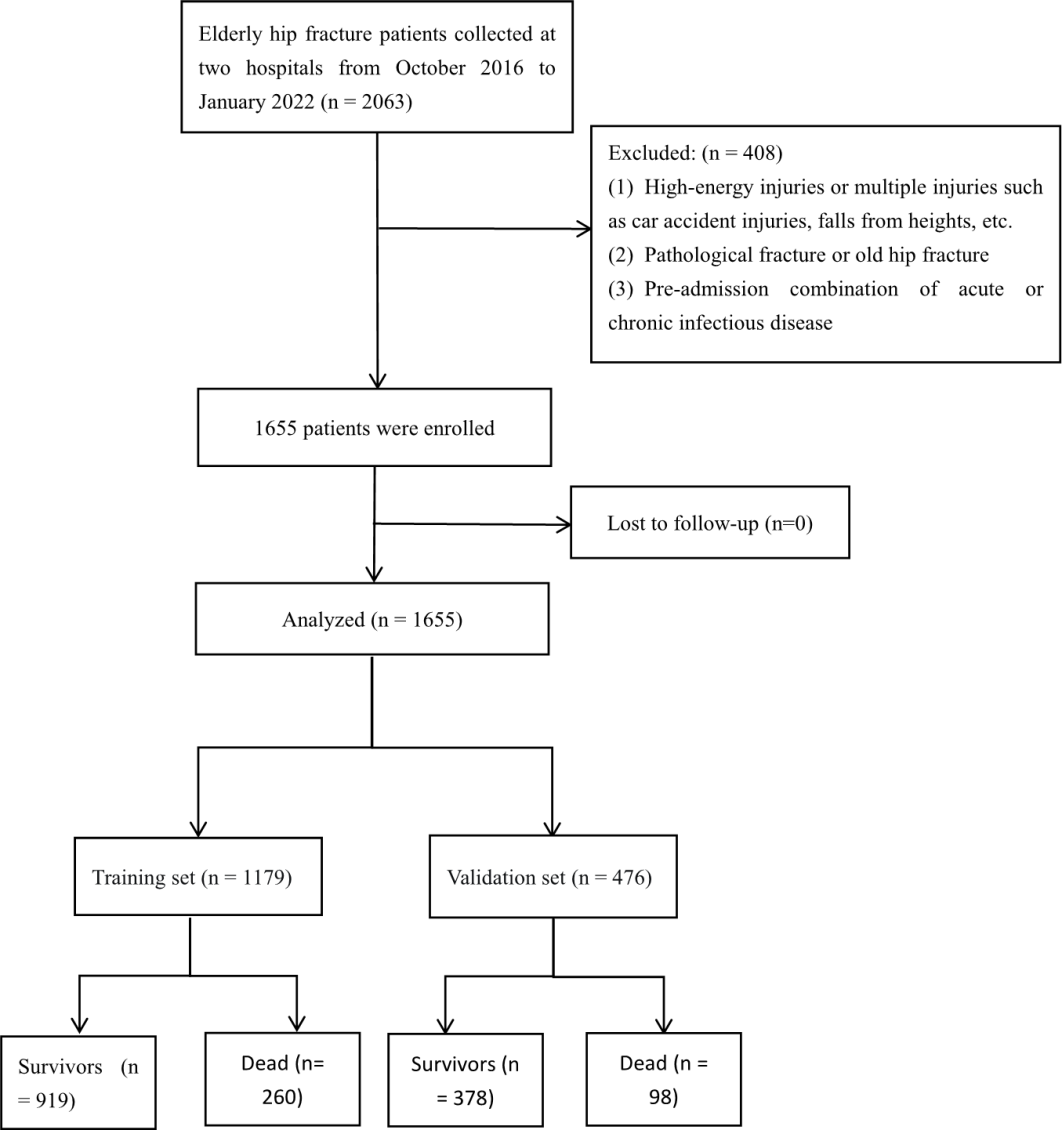
Flowchart of the enrolled patients.

This study complied with the Declaration of Helsinki. It was approved by the Ethics Committee of Guangzhou Red Cross Hospital, affiliated with Jinan University, and the Ethics Committee of Xiaogan Central Hospital, affiliated with Wuhan University of Science and Technology. Informed consent is waived with the approval of the hospital ethics committee. Data from elderly hip fracture patients were collected at two hospitals from October 2016 to January 2022, with one hospital as the training set (n=1179) and the other as the validation set (n=476). Inclusion criteria were (1): >65 years of age;(2) Unilateral hip fracture due to low-energy injury;(3) Surgical treatment (internal fixation or joint replacement);(4) The patient can walk normally before the fracture. Exclusion criteria:(1) High-energy injuries such as car accident injuries, falls from height, or multiple injuries;(2) pathological fractures or old hip fractures;(3) Pre-admission comorbid acute or chronic infectious diseases. (**Figure 1**).

The primary endpoint was defined as all-cause death. During the follow-up period, patients were evaluated by telephone and/or outpatient visit once every 3 months. Endpoint events were confirmed by medical staff and patients’ families.

### Data collection

All elderly hip fracture patients were reviewed by 2 investigators independently applying inclusion criteria. Demographic variables, clinical data, and blood tests were collected from patients at admission, and any inconsistencies were resolved by reexamining the data.

We collect patient data from electronic and paper medical records and record them on a designed record sheet at (1) Demographic variables: Age, sex, smoking, drinking, living alone. (2) Clinical data: SBP, DBP, ASA grade, time from injury to surgery, duration of surgery, length of hospital stay, intraoperative blood loss, hip fracture type, method of anesthesia, medical history (senile dementia, diabetes, COPD, coronary heart disease). (3) Blood tests: serum sodium, potassium, RDW, hemoglobin, platelets, fibrinogen, WBC, neutrophil, ESR, monocyte, CRP, IL-6, neutrophil percentage, albumin, NPAR.

### Inflammation biomarkers

Blood samples for routine blood tests and other laboratory parameters were collected from venipuncture upon admission and analyzed within 3 hours. Blood samples were tested with an XN-10 (B4) fully automated blood cell analyzer (Sysmex, Kobe, Japan) and an electrochemiluminescent immunoassay system (Roche, Cobas E411).

### Statistical analysis

Continuous data were tested for normal distribution using the Shapiro–Wilk test. Data that conformed to a normal distribution are expressed as mean and standard deviation, and those that did not are expressed as the median and interquartile range (IQR). Data for categorical variables are expressed as frequencies and proportions. Student’s t-test and Mann-Whitney U-test were used to analyze the continuous variables. The chi-square or Fisher exact test was used to compare categorical variables. Pearson correlation analysis was used to calculate the correlation coefficient matrix between inflammatory markers.

A least absolute shrinkage and selection operator (LASSO) regression model was applied to the training set for data downscaling and variable selection. The characteristic variables screened by Lasso regression were analyzed using multifactorial logistic regression analysis. To explore independent risk factors associated with 1-year all-cause mortality in elderly patients with hip fracture and to create nomograms in the training set. Receiver operating characteristic (ROC) curves were calculated to compare the diagnostic value of clinical test parameters and determine their critical importance. The concordance index (c-index), Hosmer-Lemesow goodness-of-fit test, ROC curve, calibration curve, decision curve analysis (DCA), and clinical impact curve (CIC) were used to assess the predictive accuracy of the model. Finally, the Nomogram constructed in the training set is validated using the validation set. In addition, Kaplan-Meir curves and multifactorial Cox regression analysis were used to assess the association between NPAR values and death within 1 year in geriatric hip fracture patients. The log-rank test compares the survival differences between the two groups. Results were considered statistically significant at p < 0.05. These statistical analyses were performed using SPSS 24.0, GraphPad Prism 8, MedCalc 19, and R software.

## Results

### Comparison of general clinical data of elderly hip fracture patients in the training and validation sets

**Figure 2:**
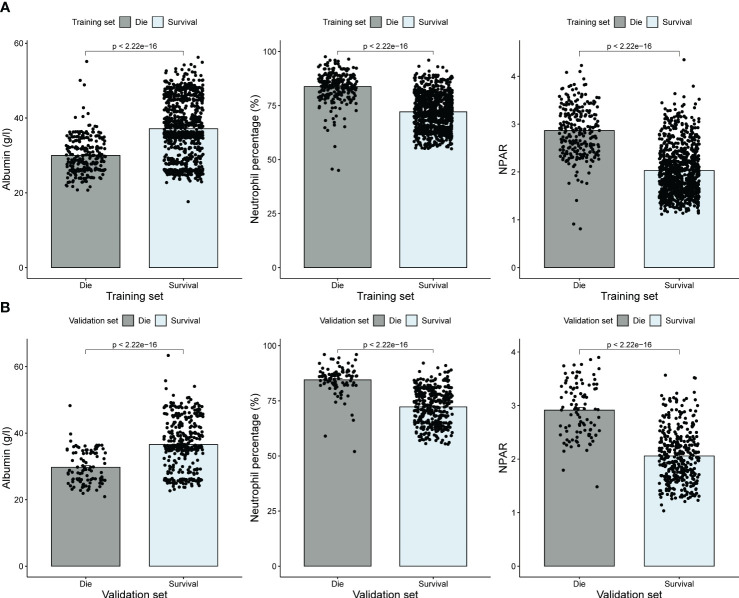
Albumin, neutrophil percentage, and NPAR levels at admission in the training set **(A)** and validation set **(B)**.

A total of 1655 patients were included in this study, including 1179 in the training set and 476 in the validation set. The mean age of the patients in the training set was 80.3 years, of which 614 were men (52.1%). The mean age of the patients in the validation set was 81.1 years, of which 220 were men (48.9%). All-cause mortality within 1 year was observed in 22.1% and 20.6% of patients in the training and validation sets, respectively. **Table 1** summarizes the clinical characteristics of the patients in the training and validation sets. The distributions of all features in the training and validation sets are almost no different. In both the training and validation sets, more patients in the death group had higher NPAR than the survivor group. This suggests that high NPAR values may be associated with poor prognosis 1 year after surgery in elderly hip fracture patients (**Figures 2A, B**).

**Table 1:** Patient characteristics of the training cohort and the validation cohort.

characteristics	Total (n = 1655)	Training set (n = 1179)	Validation set (n = 476)	P value
Demographics				
Age, year, mean(SD)	81.30 (8.13)	80.34 (8.06)	81.18 (8.33)	P = 0.267†
Sex, male, n(%)	847 (51.2%)	614 (52.1%)	233 (48.9%)	P = 0.249§
Smoking, n (%)	542 (32.7%)	383 (32.5%)	159 (33.4%)	P =0.719§
Drinking, n (%)	642 (38.8%)	456 (38.7%)	186 (39.1%)	P = 0.511§
Live alone, n (%)	885 (53.5%)	628 (53.3%)	257 (54.0%)	P = 0.789§
Clinical characteristics				
Admission SBP, mmHg, median (IQR)	150 (137-164)	150 (137-165)	150 (137-163)	P = 0.624‡
Admission DBP, mmHg, median (IQR)	98 (89-103)	98 (89-103)	98 (89-105)	P = 0.412‡
ASA grade, n (%)				P = 0.494§
II	539 (32.6%)	375 (31.8%)	155 (34.5%)	
III	1029 (62.2%)	739 (62.7%)	276 (60.9%)	
IV	87 (5.3%)	65 (5.5%)	21 (4.6%)	
Time from injury to surgery, days, median (IQR)	4 (3-6)	4 (3-6)	3 (2-4)	P < 0.001‡
Duration of surgery, minutes, median (IQR)	54 (49-60)	54 (49-60)	55 (50-60)	P = 0.338‡
Length of stay, days, median (IQR)	7 (6-9)	8 (6-9)	5 (7-9)	P = 0.532‡
Intraoperatve blood loss, ml, median (IQR)	95 (70-120)	95 (70-125)	85 (65-110)	P <0.001‡
Hip fracture type, n (%)				P = 0.298§
Neck	786 (47.5%)	550 (46.6%)	236 (49.6%)	
Intertrochanteric	797 (48.2%)	581 (49.3%)	216 (45.4%)	
subtrochanteric	72 (4.3%)	48 (4.1%)	24 (5.0%)	
Method of anesthesia, n (%)				P = 0.745§
Tracheal intubation anesthesia	831 (50.2%)	589 (50.0%)	242 (50.8%)	
Continuous epidural anesthesia	824 (49.8%)	590 (50.0%)	234 (49.2%)	
Medical history, n(%)				
Senile dementia	370 (22.4%)	277 (23.5%)	93 (19.5%)	P = 0.080§
Diabetes	823 (50.0%)	606 (51.4%)	217 (45.6%)	P = 0.032§
COPD	826 (49.9%)	594 (50.4%)	232 (48.7%)	P = 0.545§
Coronary heart disease	807 (48.8%)	565 (47.9%)	242 (50.8%)	P = 0.282§
Blood tests				
Serum sodium, mmol/l, median (IQR)	132 (120-145)	132 (119-145)	133 (120-145)	P = 0.444‡
serum potassium, mmol/l, median (IQR)	4.26 (3.43-5.12)	4.30 (3.46-5.13)	4.16 (3.33-5.00)	P = 0.024‡
Serum calcium, mmol/l, Mean(SD)	2.43 (0.40)	2.44 (0.40)	2.42 (0.39)	P = 0.868†
RDW, %, median (IQR)	14.5 (12.10-16.30)	14.5 (12.10-16.40)	14.3 (11.90-16.30)	P = 0.502‡
Hemoglobin,g/L, median (IQR)	119 (96-139)	118 (95-138)	120 (97-140)	P = 0.271‡
Platelets,10^9^/l, median (IQR)	201 (149-257)	203 (149-262)	195 (149-248)	P = 0.238‡
Fibrinogen, g/L, median (IQR),	5.32 (3.91-6.98)	5.34 (3.89-7.10)	5.29 (3.96-6.64)	P= 0.721‡
WBC, 10^9^/l, median (IQR)	11.53 (8.35-14.76)	11.62 (8.41-14.73)	11.39 (8.18-14.89)	P = 0.498‡
Neutrophil, 10^9^/L, median (IQR)	11.2 (6.3-14.9)	11.30 (6.4-15.0)	11.2 (6.2-14.9)	P = 0.347‡
ESR, mm/h, Mean(SD)	26.02 (11.30)	26.21 (11.14)	25.56 (11.14)	P = 0.094†
Monocyte,10^9^/L, median (IQR)	0.64 (0.35-0.87)	0.64 (0.35-0.88)	0.64 (0.34-0.88)	P = 0.948‡
CRP, mg/L, median (IQR)	65.30 (32.3-100.9)	66.4 (32.4-104.4)	65.2 (32.1-100.9)	P = 0.161‡
IL-6, Pg/ml, median (IQR)	121 (48-172)	122 (56-172)	122 (38-172)	P = 0.020‡
Neutrophil percentage, %, median (IQR)	75.5(67.20-82.00)	75.2(67.30-82.0)	76.05(66.2-82.0)	P = 0.859‡
Albumin, g/L, median (IQR)	36 (28-40)	36 (28-40)	35 (28-40)	P = 0.355‡
NPAR, median (IQR)	2.14 (1.74-2.62)	2.13 (1.73-2.63)	2.18 (1.75-2.62)	P = 0.436‡
Status n(%)				P = 0.513§
Survivors	1297 (78.4%)	919 (77.9%)	378 (79.4%)	
Dead	358 (21.6%)	260 (22.1%)	98 (20.6%)	

^†^Student t-test. §Pearson chi-square. ‡Mann-Whitney U test. SD, standard deviation; IQR, interquartile range; SBP, Systolic Blood Pressure; DBP, Diastolic Blood Pressure; ASA, American Society of Anesthesiologists Physical Status Classification; COPD, chronic obstructive pulmonary disease; RDW, red cell distribution width; WBC, white blood cell; ESR, Erythrocyte sedimentation rate; CRP, C-reactive protein; IL-6, Interleukin-6; NPAR, neutrophil percentage-to-albumin ratio.

### Lasso regression analysis for risk factor selection

**Figure 3:**
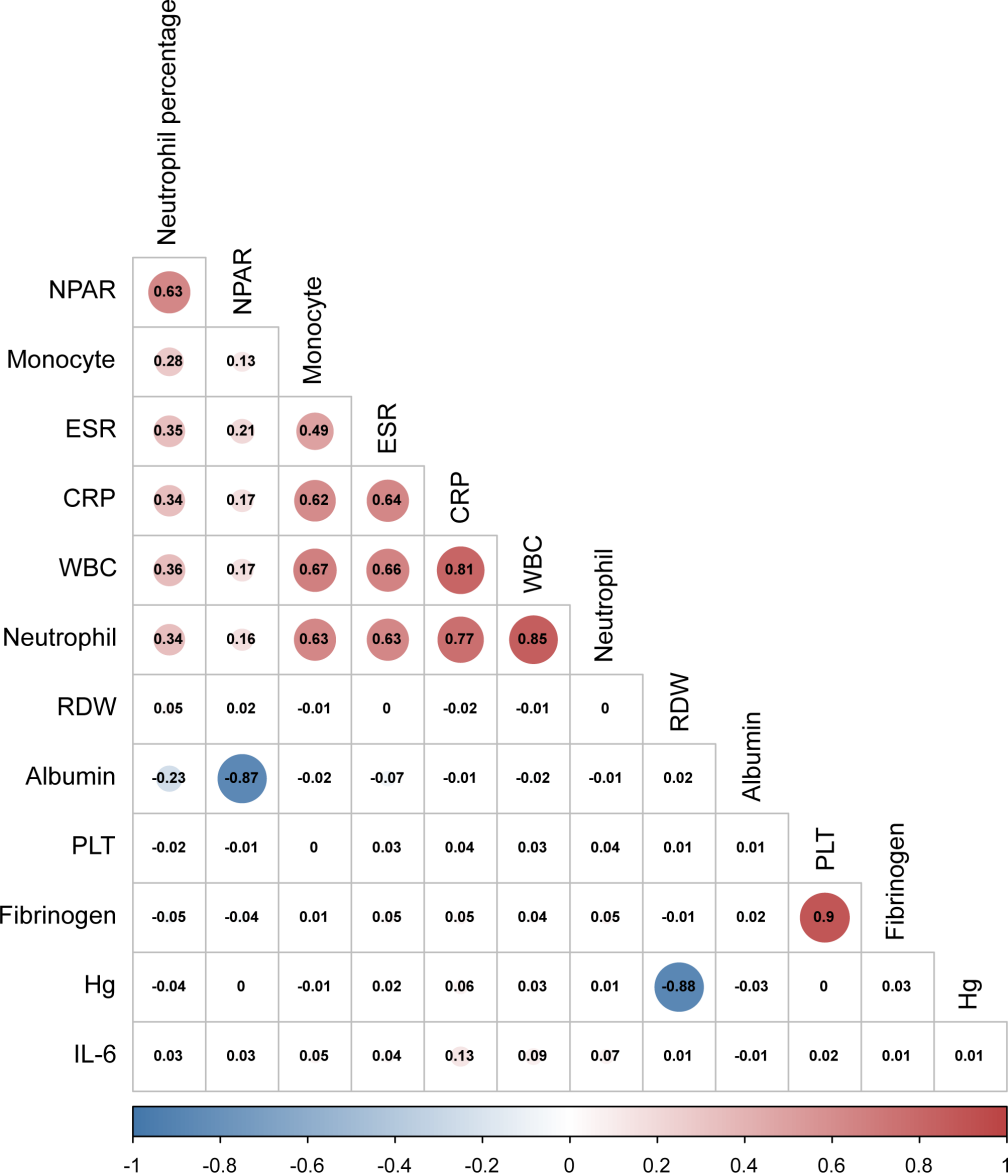
Pairwise Pearson correlation coefficients among inflammatory markers. Blue indicates a positive correlation, and red indicates a negative correlation. Darker colors are associated with stronger correlation coefficients.

**Figure 4:**
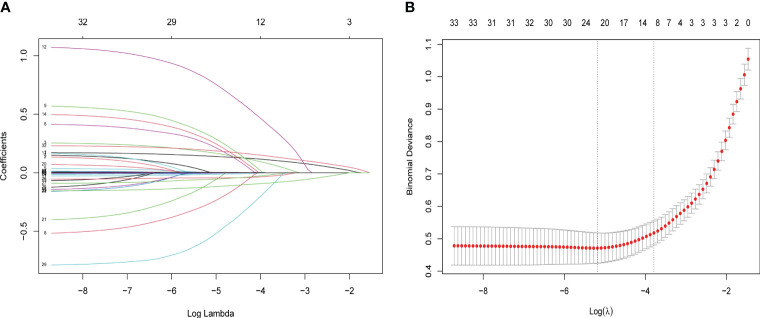
LASSO regression analysis performed feature selection on candidate variables using a 10-fold cross-validation method. **(A)** Coefficient profile diagram. Each curve in the figure represents the change trajectory of each independent variable coefficient. The ordinate is the value of the coefficient, and the abscissa is the number of non-zero coefficients in the model. With the constant value of the penalty parameter lambda increasing, the final variable coefficient gradually approaches 0. **(B)** The left dashed line corresponds to the l value with the smallest mean square error, and the right dashed line corresponds to the l value of the simplest model within a variance range of the minimum l value.

The Pearson correlation method was used to calculate the correlation coefficients of inflammatory markers, and the results showed a high correlation between inflammatory markers (**Figure 3**). Therefore, to select independent risk factors, Lasso regression analysis was performed on all variables in the training set to identify factors associated with poor prognosis 1 year after hip fracture in older people. Finally, we selected the nine variables with the best lambda: age, time from injury to surgery, senile dementia, WBC, monocyte, neutrophil, neutrophil percentage, and NPAR (**Figures 4A, B**).

### Multivariable logistic regression to select risk factors

**Figure 5:**
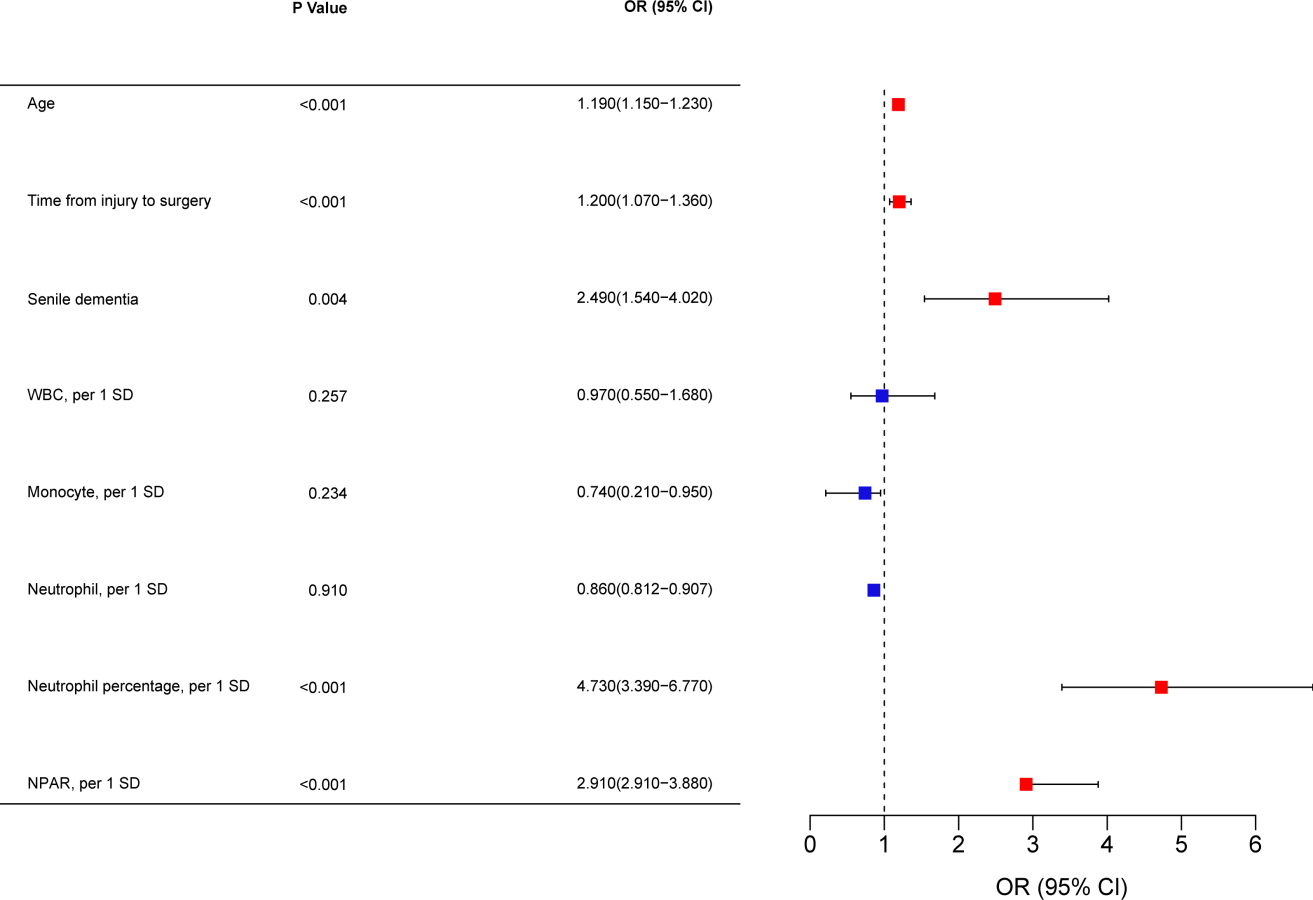
A multivariate logistic regression analysis. OR, odds ratio; CI, confidence interval; WBC, count of white blood cells; NPAR, neutrophil percentage-to-albumin ratio; SD, standard deviation.

To identify independent risk factors predicting poor 1-year prognosis in elderly patients with hip fracture, multivariable logistic regression analysis was used further to screen the results of Lasso regression for predictors. Finally, the following variables were significantly associated with poor patient prognosis at one year: age (OR= 1.190,95% Cl= 1.150-1.230, P< 0.001), time from injury to surgery (OR= 1.200,95% Cl= 1.070-1.360, P< 0.001), senile dementia (OR= 2.490,95% Cl= 1.540-4.020, P= 0.004), neutrophil percentage (OR= 4.730,95% Cl= 3.380-6.790, P< 0.001), NPAR (OR= 2.920,95% Cl= 2.910-3.880, P< 0.001) (**Figure 5**).

### ROC curves for each factor in the multivariable logistic regression model

**Figure 6:**
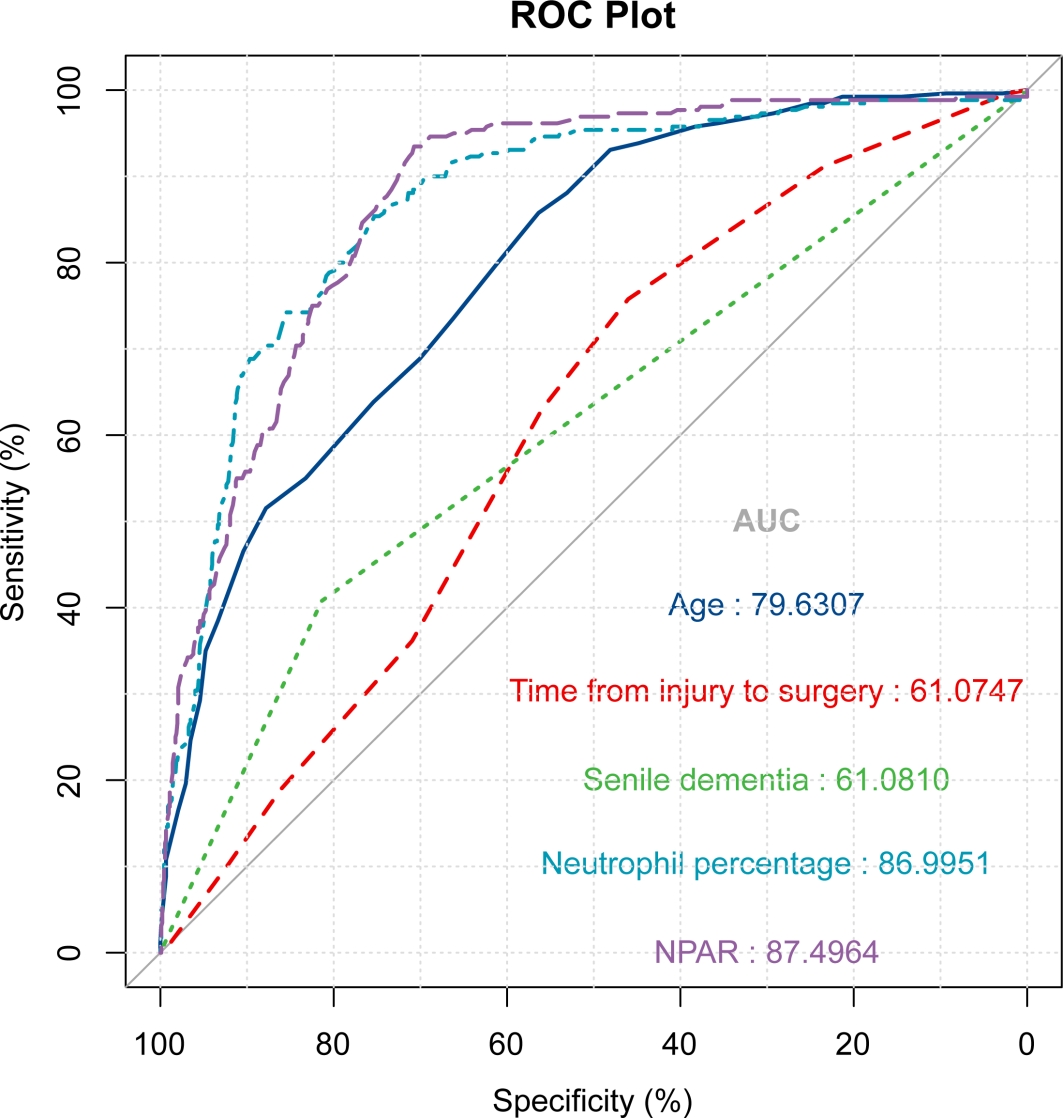
ROC curves of the multivariate logistic regression analysis results in the training set.

**Figure 7:**
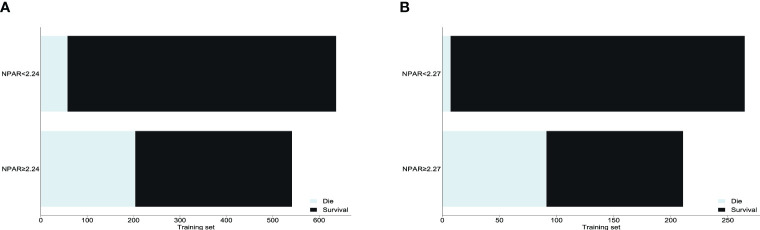
**(A)** Distribution of patients with different prognoses in training set grouped by NPAR cut-off values. **(B)** Distribution of patients with different prognoses grouped by NPAR cut-off values in the validation set. NPAR, neutrophil percentage-to-albumin ratio.

In the training set, variables with P<0.05 in the multivariate logistic regression analysis were included in the ROC curves (**Figure 6**, **Table 2**). Among the risk factors predicting poor prognosis of patients at 1 year after surgery, the AUC for NPAR was significantly higher than that for age (P<0.001), time from injury to surgery (P<0.001), and senile dementia (P<0.001), and comparable to the AUC for neutrophil percentage (P=0.128). The receiver operating characteristic curve (ROC) was used to determine the optimal cut-off value for NPAR.In the training set, we divided the patients into two groups with a cut-off value of 2.24 for NPAR as the node. We analyzed the distribution of patients with different prognoses 1 year after surgery in the two groups. The results showed that a higher proportion of patients survived within 1 year in the NPAR<2.24 group. The ratio of patients who died within 1 year to those who survived was comparable in the NPAR ≥2.24 group (**Figure 7A**). In the validation set, we divided the patients into two groups with a cut-off value of 2.27 for NPAR as the node. We analyzed the distribution of patients with different prognoses 1 year after surgery in the two groups. The results showed that the proportion of patients surviving within 1 year was significantly higher in the NPAR<2.27 group than in the NPAR≥2.27 group (**Figure 7B**).

**Table 2:** Comparison of AUC of NPAR with other influencing factors.

	AUC	95% Cl	P Value
Age	0.796	0.772 - 0.819	P<0.001
Time from injury to surgery	0.642	0.614 - 0.670	P<0.001
Senile dementia	0.611	0.582 - 0.639	P<0.001
Neutrophil percentage	0.870	0.849 - 0.889	P=0.128
NPAR	0.875	0.855 - 0.893	

AUC, an area the under curve; CI, confidence interval; NPAR, neutrophil percentage-to-albumin ratio.

### Nomograms and Bootstrap validation of the multivariable logistic regression models

**Figure 8:**
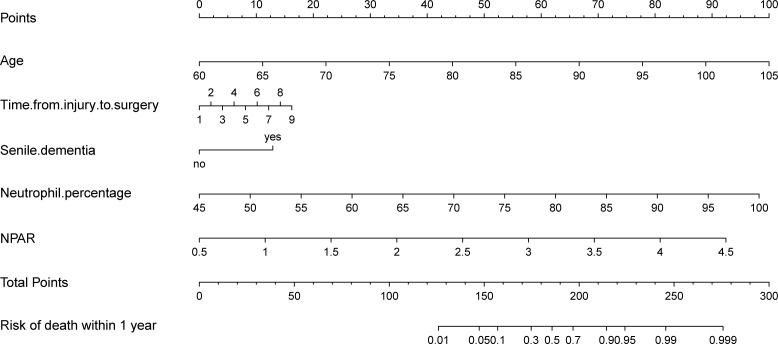
Nomogram model for predicting all-cause mortality in elderly hip fracture patients 1 year after surgery.

In the training set, five variables (age, time from injury to surgery, senile dementia, neutrophil percentage, and NPAR) were selected for multivariate logistic regression analysis to construct Nomograms predicting poor 1-year prognosis in elderly hip fracture patients and validated by Bootstrap (**Figure 8**).

When NPAR was included, the c-index was 0.942(95% CI:0.926-0.956); when NPAR was excluded, the c-index decreased to 0.927(95% CI:0.909-0.941). This indicates that the multivariable logistic regression model, including NPAR, had good consistency with the actual situation and had better assessment efficacy.

### ROC curves and calibration curves of the multivariable logistic regression models

**Figure 9:**
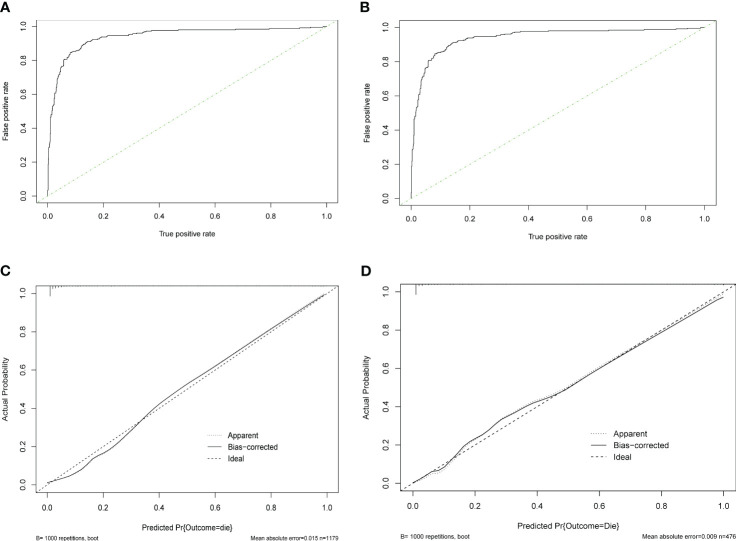
ROC curves are used to evaluate the prediction effect of **(A)** training set and **(B)** validation set. **(C)** Calibration curves for assessing prognosis in the training set; **(D)** for evaluating prognosis in the validation set.

In the training set, the area under the ROC curve of the evaluated model was 0.942(95% CI:0.927-0.955 (**Figure 9A**). When NPAR was excluded, the area under the ROC curve was 0.927(95% CI:0.910-0.941). This indicates that the NPAR model has high sensitivity and specificity and is a better prediction model. The calibration curves revealed good predictive accuracy of the nomograms (**Figure 9C**). The Hosmer-Lemesow goodness-of-fit test showed that the model agreed well in assessing the poor prognosis of elderly hip fracture patients 1 year after surgery (P>0.05).

In the validation set, the area under the ROC curve of the evaluated model was 0.965 (95% CI: 0.944-0.980) (**Figure 9B**). This indicates that the NPAR model has high sensitivity and specificity and is a better prediction model. The calibration curves revealed good predictive accuracy of the nomograms (**Figure 9D**). The Hosmer-Lemesow goodness-of-fit test showed that the model agreed well in assessing the poor prognosis of elderly hip fracture patients 1 year after surgery (P>0.05).

### Decision curves and clinical impact curves for multivariate logistic regression models

**Figure 10:**
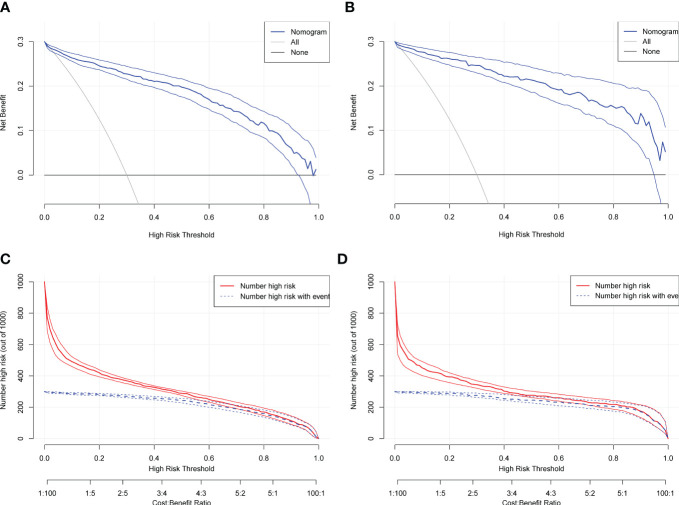
**(A)** Decision curve analysis for assessing prognosis in a training set; **(B)** Decision curve analysis for assessing prognosis in a validation set. **(C)** Clinical impact curves for prognosis assessment in the training set; **(D)** Clinical impact curves for prognosis assessment in the validation set.

Prediction models for multiple independent risk factors, such as NPAR, were constructed in the training and validation sets. The applicability and validity of the model were evaluated by decision curve analysis (DCA). The results show that this model provides a significant additional net clinical benefit to patients in predicting poor prognosis 1 year after hip fracture surgery in older adults., with good clinical applicability and effectiveness (**Figures 10A, B**). The clinical impact curve (CIC) results showed that the two curves converged after a risk threshold of 0.5 (training set) and 0.6 (validation set) (**Figures 10C, D**). This model has some clinical applicability and validity in predicting poor prognosis 1 year after hip fracture surgery in older adults.

### Multivariate Cox regression analysis and Kaplan-Meier survival curve analysis

**Figure 11:**
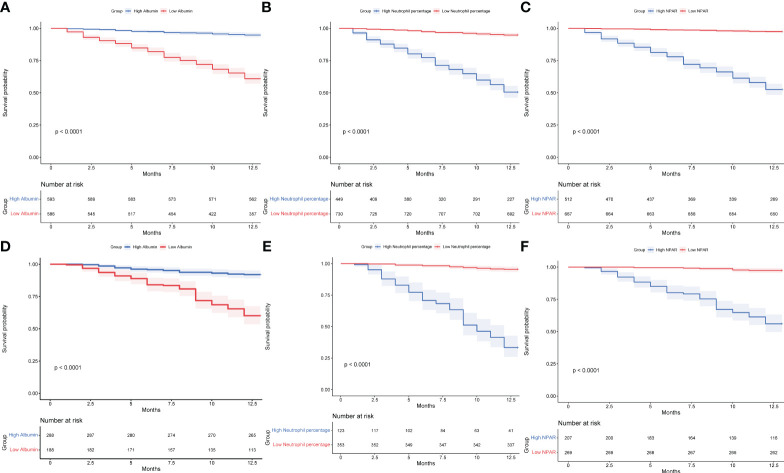
Kaplan-Meier curves of patients in the high ratio and low ratio groups in the training set, **(A)** albumin; **(B)** neutrophil percentage; **(C)** NPAR. Kaplan-Meier curves of patients in the high ratio and low ratio groups in the validation set, **(D)** albumin; **(E)** neutrophil percentage; **(F)** NPAR.

**Figure 12:**
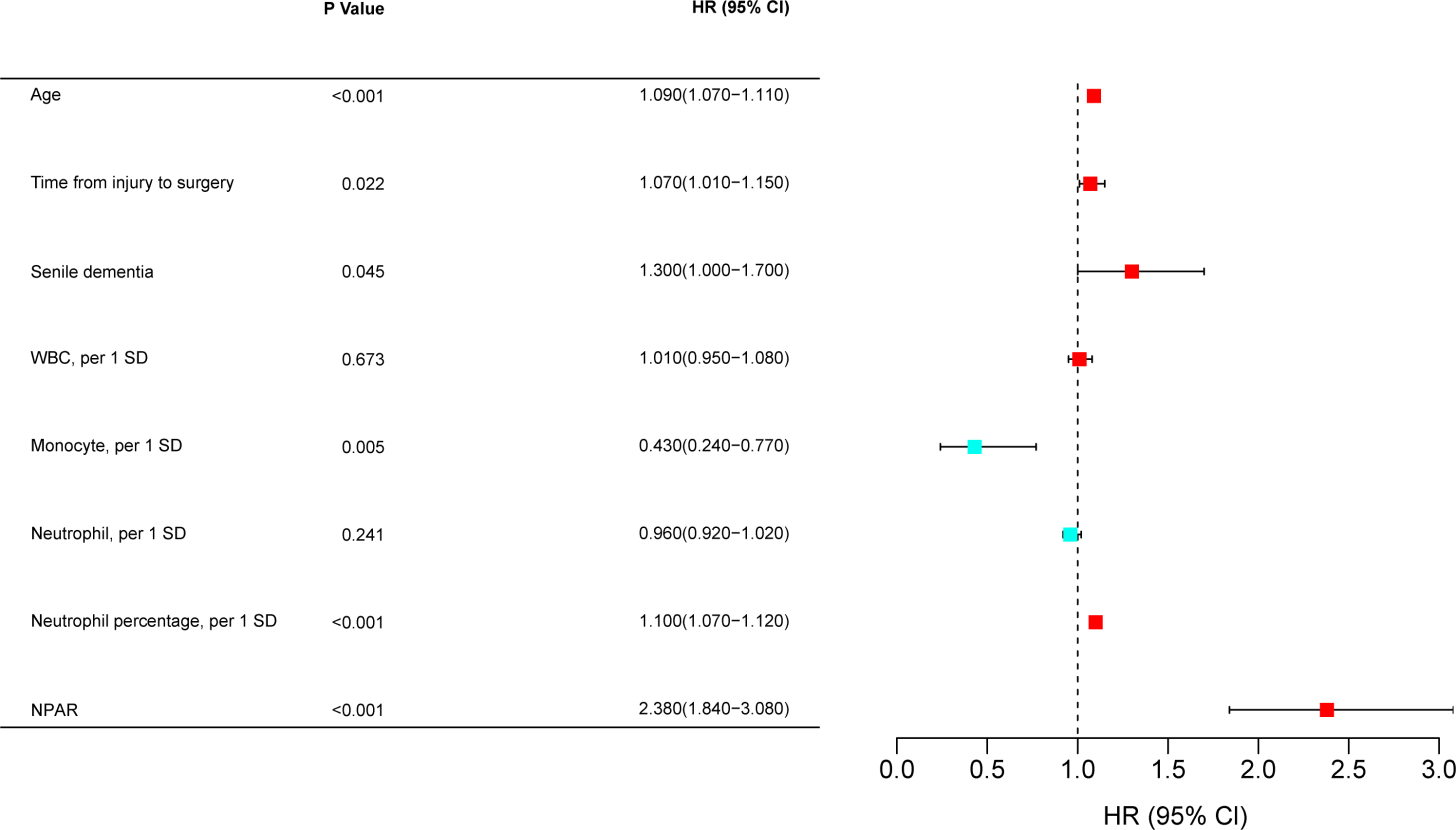
Multivariate Cox regression analysis. HR, hazard ratio; CI, confidence interval; WBC, count of white blood cells; NPAR, neutrophil percentage-to-albumin ratio; SD, standard deviation.

In the training set, factors screened by Lasso regression were included in multivariate Cox analysis, which showed that age (HR= 1.090,95% Cl= 1.070-1.110, P< 0.001), time from injury to surgery (HR= 1.070,95% Cl= 1.010-1.150, P= 0.022), senile dementia (HR= 1.300,95% Cl= 1.000-1.700, P= 0.045), neutrophil percentage (HR= 1.100,95% Cl= 1.070-1.120, P< 0.001), and NPAR (HR= 2.380,95% Cl= 1.840-3.080, P< 0.001) as 1-year independent risk factor for death (**Figure 11**). In both the training and validation sets, Kaplan-Meier survival curves showed that patients in the high NPAR value group and the high ratio of neutrophil percentage group had a higher mortality rate at 1 year (P< 0.001); the low albumin patient group had a higher mortality rate at 1 year (P< 0.001) (**Figure 12**).

## Discussion

Hip fractures are a common problem for aging societies worldwide (18). Patients with hip fractures are at high risk of death, with mortality rates as high as 15-36% at 1 year postoperatively (2–4). Predicting the risk of death in hip fracture patients in advance and giving treatment can help improve the prognosis. However, there is a lack of biological indicators that can better assess the poor prognosis of elderly hip fractures 1 year after surgery. Therefore, this study sought to develop a new mortality risk screening tool, NPAR, and explore its role in assessing poor prognosis at 1 year postoperatively.

This study retrospectively analyzed the clinical data of 1583 hospitalized patients, divided into a training set and an external validation set. The all-cause mortality rate of patients 1 year after surgery was 22.4%, consistent with other reports in the literature (2, 3). Considering the possibility of multicollinearity in inflammatory biomarkers, we used the least absolute shrinkage and selection operator (LASSO) analysis and multivariate logistic regression to select variables. Five independent risk factors were identified in the training set, and a Nomogram model was built. The clinical usefulness of the model was demonstrated using an external validation set, and the results indicate that our model has good clinical application.

Age is an important factor affecting the postoperative prognosis of elderly hip fracture patients (18, 19). Most elderly patients undergoing surgery and anesthesia experience a significant decline in physical function and an increased risk of postoperative death (20, 21). This is consistent with our results. The results showed that age was an independent risk factor for poor prognosis 1 year after hip fracture surgery in older adults. However, the results of Jacques’ study (22) concluded that age did not affect the survival of patients after surgery, after excluding the interference of underlying medical disease. The impact of the prolonged time from injury to surgery on the postoperative prognosis of elderly hip fracture patients is directly reflected in the increased time that patients spend in bed, which contributes to a significantly higher incidence of bedridden complications and affects late recovery (23). Rosso et al. (24) recommended that patients with hip fractures undergoing surgery within 48 h may reduce mortality within 1 month after surgery. Cha et al. (23) found that delayed surgery was significantly associated with 30-d and 1-year mortality in a retrospective study. This showed the same results in our research. However, other scholars believe surgery should not be rushed and that sufficient time should be allowed to treat medical conditions in elderly patients before surgery (25). At the same time, preoperative preparation and evaluation should be improved to reduce the risk of surgery and improve the success rate. Vidal et al. (26) concluded that the time between injury and surgery was not associated with in-hospital mortality and mortality at 1 year postoperatively in patients with fragility hip fractures. However, the preoperative surgery time control must be carefully decided after individual analysis of the patient’s status (27). Systematic preoperative evaluation and management of the condition will help to ensure a safe and smooth operation. Some studies have reported that senile dementia is classified into different levels of severity, and the more severe the level, the worse the prognosis, even leading to the patient’s death (28). Tarazo⁃na et al. (29)found that patients with different degrees of cognitive impairment, especially in the mild and moderate groups, had similar mortality rates within six months of surgery despite poorer functional status and more complications. Mortality within 1 year increases with the severity of the cognitive impairment.

Serum albumin is a plasma protein with various roles, including regulating inflammation, maintaining normal colloid osmotic pressure, and transporting many substances (30). Also, albumin can affect the pharmacokinetics of many drugs, thus affecting their efficacy. Previous studies have found that low albumin levels are associated with increased cardiovascular disease or mortality (31). Related studies have found that low Alb levels have a poor prognosis and that patients’ preoperative Alb levels positively correlate with postoperative recovery (30). This is in general agreement with the results of our study. In this study, Alb levels were higher in patients in the survivor group compared to the death group, suggesting that Alb levels correlate with patients’ postoperative prognostic status.

With the current understanding of fracture pathophysiology, the role played by the inflammatory response in the development of fractures is gradually gaining attention from scholars (32). Neutrophils are important in mediating the inflammatory response as essential components of leukocytes and key effector cells. Also, neutrophils are an important component of the innate immune system (10). Lymphocytes, another common leukocyte subtype, are the most critical inflammatory trigger for activating the adaptive immune response (33). Neutrophil percentage not only directly reflects the ratio of neutrophils but also indicates the ratio of lymphocytes to a certain extent. In our study, we found a higher percentage of neutrophils in the patients in the death group than in the surviving group, suggesting that higher levels of neutrophil percentage may be associated with poor postoperative prognosis.

NPAR is a recently identified potent biomarker of systemic infection and inflammation (34). NPAR combines neutrophil percentage and albumin levels, integrating different factors of inflammation and immune response, which may make it a more representative biomarker. Changes in NPAR better reflect the dynamic balance of immunity and inflammation and disease activity, making it an ideal biological indicator. Recent studies have shown that elevated NPAR levels are strongly associated with poor prognosis in patients with cardiovascular disease (35). In patients with acute myocardial infarction, higher NPAR values were related to short-term mortality (36). In this study, patients in the death group had higher NPAR levels than the survivor group, suggesting that NPAR levels correlate with patients’ postoperative prognosis. Also, we determined the optimal cut-off value of NPAR based on the receiver operating characteristic (ROC) curve. We found a higher proportion of surviving patients in the low ratio group, suggesting that the lower the NPAR value, the better the prognosis of the patients after surgery. Among the prediction models constructed from multiple independent risk factors, the AUC of the ROC curve of the model, including NPAR, was more significant, with good discrimination and consistency. The Nomogram model with the NPAR removed has a lower C-index and reduced evaluation power. ROC curves were plotted for comparative analysis of each risk factor, and the results showed that the AUC of NPAR was the largest among multiple independent risk factors. The present results suggest that NPAR is a good indicator with good sensitivity and specificity for assessing the postoperative prognosis of elderly hip fracture patients. In the training and external validation sets, we use calibration curves and Hosmer-Lemesow goodness-of-fit tests to evaluate the calibration ability of the model. In addition, we used decision curve analysis and clinical impact curves to evaluate the clinical benefits of the model. The results showed that the model has good clinical applicability and validity.

Although the possible role of NPAR in the prognosis of patients with hip fractures has not been elucidated in basic trials, several hypotheses explain the predictive value of NPAR. Neutrophils mediate the inflammatory response as important components of leukocytes and key effector cells (10). Hip fractures can lead to a severe systemic inflammatory response, which increases the proportion of neutrophils. At the same time, when a hip fracture occurs in the senior, inflammatory mediators act on the body’s tissues and organs (such as the liver) to damage them. The energy consumption of the damaged tissues and organs, especially the liver cells, increases, their ability to synthesize and secrete albumin decreases, and the concentration of albumin in the blood decreases. In addition, patients with hip fractures are in a hypermetabolic state, which is characterized by accelerated proteolytic metabolism and decreased anabolism, resulting in a persistent negative nitrogen balance (37). In addition, previous clinical practice has found (38) that geriatric hip fracture patients are often admitted to the hospital in a state of malnutrition, mainly associated with reduced metabolic function in elderly patients. The NPAR value combines neutrophil percentage and albumin levels and can represent a composite index of inflammatory, malnourished conditions.

Individualized and timely risk assessment of each hip fracture patient allows for more accurate decisions about treatment strategies and allocation of medical resources. In contrast to relatively complex clinical scores, the NPAR appears to be an easy-to-use outcome stratification biomarker that can be easily obtained from routine blood test results and thus can be used as a valid marker for rapid risk assessment. Clinical workers can stratify the management of patients based on their NPAR values at the time of admission. Patients with hip fractures with high NPAR values should be of greater concern to clinicians. Prompt preoperative albumin supplementation in patients with high NPAR values and effective control of the inflammatory response reduces the risk of death in patients within 1 year after surgery.

Our research has some advantages. First, our research had a relatively large sample size and combined patient information from two regional hospitals to reduce selection bias. Second, to our knowledge, this study demonstrates for the first time the relationship between NPAR, a marker of inflammation, and the postoperative prognostic profile of elderly hip fracture patients. The NPAR values are simple to calculate, economically efficient, and can be implemented even in areas with poor medical conditions. More importantly, NPAR acts as a combination of Alb and neutrophil percentage, which may reflect, to some extent, the balance between anti-inflammatory and pro-inflammatory effects *in vivo*. Finally, we validated the prediction model in various aspects to ensure the validity and credibility of the results. However, there are several limitations to this study. First, this study is retrospective, and a prospective multicenter study is needed to confirm our conclusions further. Secondly, this study did not consider the different surgical treatment modalities of elderly hip fracture patients, such as the significantly different surgical levels of internal fixation treatment versus joint replacement options. Therefore, there may be some difference in the damage caused to the patient. We next demonstrate our conclusions by comparing patients’ surgical approaches in a stratified manner. Finally, this study was conducted in China, and it is not clear that the results would be the same in non-Asian populations and among different ethnic groups. In future studies, we will work on multiple countries and regions to validate our findings in the context of a multicenter study with a stratified analysis of racially and ethnically diverse populations.

## Conclusion

This study showed that NPAR levels were significantly higher in patients who died within 1 year after surgery. Also, NPAR was an independent predictor of poor prognosis in elderly patients with hip fractures up to 1 year after surgery.

## Data availability statement

The raw data supporting the conclusions of this article will be made available by the authors, without undue reservation.

## Ethics statement

The studies involving human participants were reviewed and approved by the Ethics Committee of Guangzhou Red Cross Hospital, affiliated with Jinan University, and the Ethics Committee of Xiaogan Central Hospital, affiliated with Wuhan University of Science and Technology. Informed consent is waived with the approval of the hospital ethics committee. Written informed consent for participation was not required for this study in accordance with the national legislation and the institutional requirements.

## Author contributions

SJ wrote the main manuscript, analyzed the data, and prepared the tables and figures. JZ, ZF, ZL, JH, and LC collected the data. QM designed the research, led the research group, and arranged the work of all authors. All authors reviewed the manuscript. All authors contributed to the article and approved the submitted version.
